# Intraoperative parathyroid hormone measurement pitfalls: parathyroid hormone spikes with carboxyl-terminal parathyroid hormone fragments in primary hyperparathyroidism—a case report

**DOI:** 10.1186/s40792-024-01903-z

**Published:** 2024-04-25

**Authors:** Hiroyuki Yamashita, Hisakazu Shindo, Kouichi Yoshimoto, Yusuke Mori, Takashi Fukuda, Seigo Tachibana, Hiroshi Takahashi, Shinya Sato

**Affiliations:** 1 Department of Surgery, Yamashita Thyroid Hospital, 1-8 Simo-Gofukumachi, Hakata-Ku, Fukuoka City, Fukuoka 812-0034 Japan; 2 Department of Endocrinology, Yamashita Thyroid Hospital, Fukuoka City, Japan

**Keywords:** Hyperparathyroidism, Intraoperative parathyroid hormone measurement, Carboxyl-terminal PTH fragment

## Abstract

**Background:**

Intraoperative parathyroid hormone (IOPTH) monitoring is a critical surgical adjunct for determining the extent of surgery for primary hyperparathyroidism (PHPT), with reported false-positive and false-negative rates of up to 10%. Surgeons must understand the parathyroid hormone (PTH) dynamics and select the appropriate IOPTH protocol and interpretation criteria for curative surgery.

**Case presentation:**

We present the case of a 64-year-old woman with a large cystic parathyroid tumor and PHPT who experienced a significant delay in IOPTH decrease but was cured without additional surgery. The patient’s basal intact PTH was 96.2 pg/mL, which decreased to 93.3 pg/mL at 25 min and 72.4 pg/mL at 55 min after removal of the parathyroid tumor. In an attempt to elucidate its pathophysiology, 1–84 PTH levels were measured in stored serum. These results can also be attributed to the relatively low basal PTH levels, intact PTH spike, and high ratio of large carboxyl-terminal PTH fragments present. The patient had normal intact PTH and calcium levels at the 9-month postoperative visit.

**Conclusions:**

As detailed reports on these phenomena are scarce, we discuss the causes of false-negative IOPTH results in terms of PTH production, secretion, metabolism, and differences in measurement methods to avoid unnecessary surgery.

## Background

Primary hyperparathyroidism (PHPT) is characterized by excessive parathyroid hormone (PTH) secretion, causing serum hypercalcemia, osteoporosis, and ureteral calculi. Ninety percent of parathyroid cases result from a single parathyroid adenoma, with less common causes including multiple hyperplasia (6%), double adenoma (4%), and parathyroid carcinoma (< 1%) [1]. Intraoperative parathyroid hormone (IOPTH) monitoring is vital for guiding surgery extent in PHPT patients. Accurate interpretation of PTH level changes is crucial for optimal curative procedures. Surgeons should grasp PTH dynamics, choose IOPTH protocols wisely, and apply careful interpretation criteria. This enhances the accuracy of predicting surgical success, minimizes unnecessary bilateral extractions, lowers the risk of resecting non-hypersecretory glands, and prevents recurrence. Despite numerous reported criteria for IOPTH, none are flawless, occasionally resulting in rare false-positive or false-negative outcomes [2–5]. A false-positive result may necessitate reoperation, whereas a false-negative result may prompt further evaluation of the pathological parathyroid glands.

We present the case of a woman with a large cystic parathyroid tumor and PHPT who experienced a delay in the decrease of intraoperative intact parathyroid hormone (IOiPTH) but was cured without additional surgery. This was attributed to a suspected iPTH spike with a high ratio of large carboxyl-terminal PTH fragments (C-PTH fragments) during the resection of a large cystic parathyroid tumor, supported by an almost certain preoperative localization diagnosis of single-gland disease.

## Case presentation

A 65-year-old woman was referred to our hospital from a thyroid clinic with thyroid tumors and hypercalcemia. She was on thyroxine for hypothyroidism due to chronic thyroiditis and had been treated for diabetes, hypertension, and hyperlipidemia; however, she had no family history of hypercalcemia. The patient, previously evaluated for hypercalcemia at another hospital, showed inconclusive 24-h urinary calcium excretion rate results for distinguishing PHPT from familial hypocalciuric hypercalcemia (FHH). Given mild hypercalcemia without a localized parathyroid adenoma and uncertainty about excluding FHH, follow-up ensued without genetic testing. The patient, lacking specific PHPT symptoms, had a 4 × 3 cm oval mass palpated in the left lower pole of the thyroid gland, with normal additional physical findings.

### Diagnostic assessment

The patient’s serum calcium was 2.75 (reference, 2.2–2.5) mmol/L, with a normal serum phosphate level of 1.13 (reference, 0.87–1.49) mmol/L. Her fractional urine excretion of calcium was 1.4%, indicating a higher probability of PHPT than of FHH. The renal function test results, complete blood count, and serum and urine electrophoresis results were normal. Her glycated hemoglobin level was 7.2% (reference, 4.6–6.2%), serum iPTH was 96.2 pg/mL ([10.2 pmol/L]; reference, 15–65 pg/mL), and bone-specific alkaline phosphatase and tartrate-resistant acid phosphatase-5b levels were 19.1 (reference, 3.8–22.6) µg/mL and 516 (reference, 120–420) mU/dL, respectively. iPTH levels were determined using an electrochemiluminescence immunoassay (COBAS 8000 e801 analyzer; Roche Diagnostics, Indianapolis, IN, USA). Additionally, serum 25 hydroxyvitamin D and 1,25-dihydroxyvitamin D levels were 26.5 (reference, > 30) ng/mL and 85.4 (reference, 20–60) pg/mL, respectively.

**Fig. 1:**
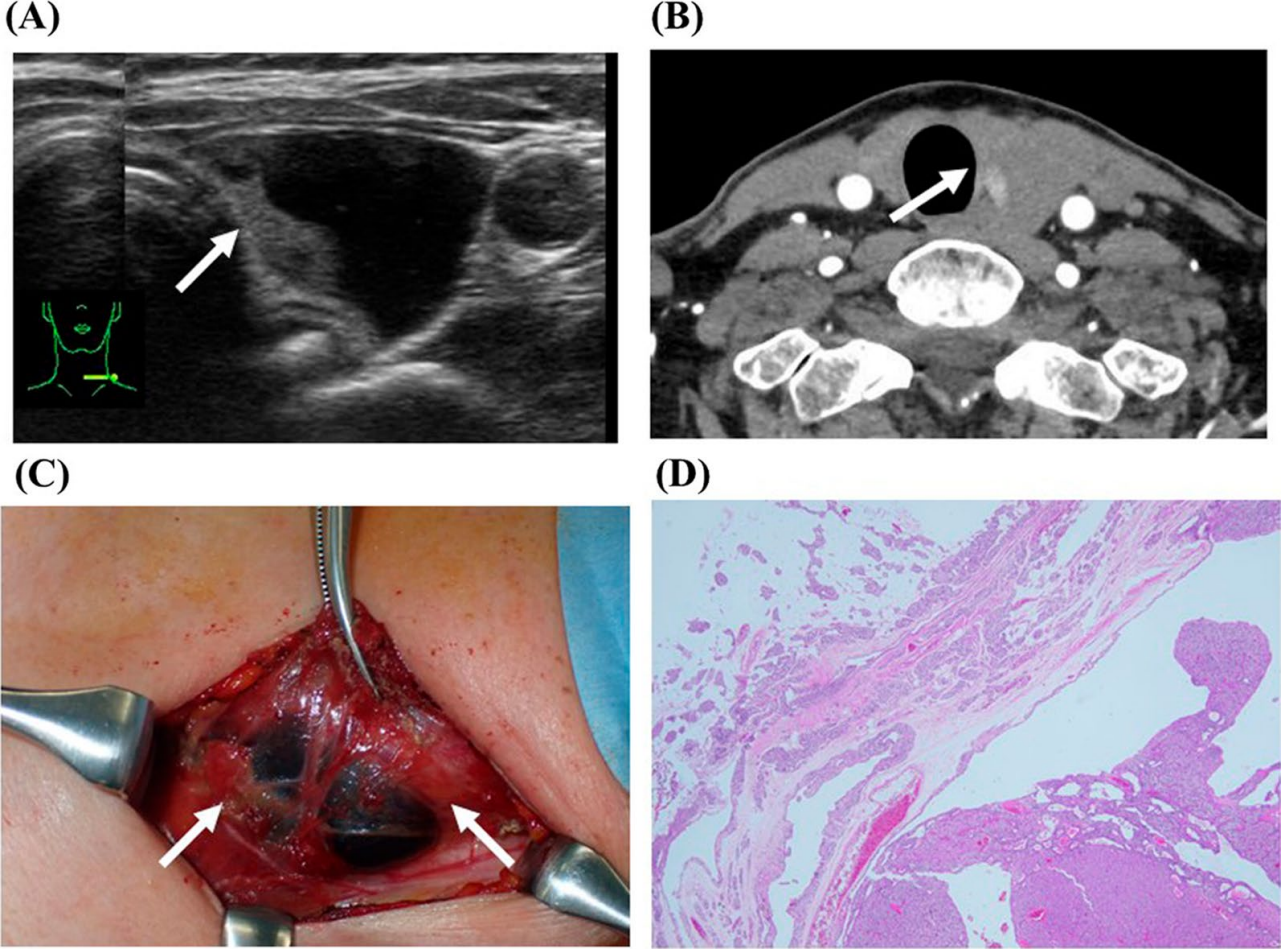
Imaging findings. **A** Ultrasonography showed a cystic nodular lesion measuring 4.7 × 2.7 × 2.3 cm. It was unclear whether the lesion was located within or outside the left lobe of the thyroid gland. **B** Contrast-enhanced computed tomography scan showing an enhanced area (white arrow) of the cystic tumor with undeniable findings of cystic parathyroid adenoma. **C** A 1.4-g parathyroid adenoma is attached to the left lobe of the thyroid gland. **D** Parathyroid tissue is composed of clear, follicular, cystic, and follicular cells of various sizes and is diagnosed as a parathyroid adenoma

Ultrasonography showed a 4.7 × 2.7 × 2.3 cm cystic nodular lesion (Fig. 1A); the lesion’s location within or outside the left lobe of the thyroid gland was unclear, and no typical enlarged parathyroid gland was observed. 99mTc-methoxy-isobutyl-isonitrile scintigraphy (99mTc-MIBI) was not performed at our hospital due to negative results elsewhere. Contrast-enhanced computed tomography (CT) scan showed a similarly sized enhancing area of the cystic tumor (Fig. 1B) with undeniable findings of cystic parathyroid adenoma. The iPTH level in the cystic fluid was very high (> 5000 pg/mL), leading to cystic parathyroid adenoma diagnosis. Bone mineral density remained preserved (lumbar spine: T-score − 1.8 SD; forearm bone: T-score 0.1 SD). Despite this, the patient opted for surgery due to neck tenderness associated with her large cystic parathyroid adenoma. Testing for MEN was not performed given the absence of MEN-related criteria, such as age < 30 years, a history or imaging suggestive of multigland disease, and/or a family history of hypercalcemia and/or syndromic disease [6].

### Treatment

Intraoperatively, a 1.4-g parathyroid adenoma attached to the left lobe of the thyroid gland was removed (Fig. 1C and D). The IOiPTH levels exhibited minimal variation, persisting nearly unchanged from 96.2 pg/mL preoperatively to 93.3 pg/mL at 25 min after removal. Suspecting a potential iPTH spike in the cystic parathyroid adenoma, an additional 30-min wait was decided. Subsequently, the iPTH level dropped to 72.3 pg/mL, falling short of curative criteria. However, the surgery was concluded without the need for reoperation. In the ward, 3 h post-adenomectomy, the iPTH reached a curative level of 24.8 pg/mL. The pathological diagnosis was cystic parathyroid adenoma.

### Outcome and follow-up

**Fig. 2:**
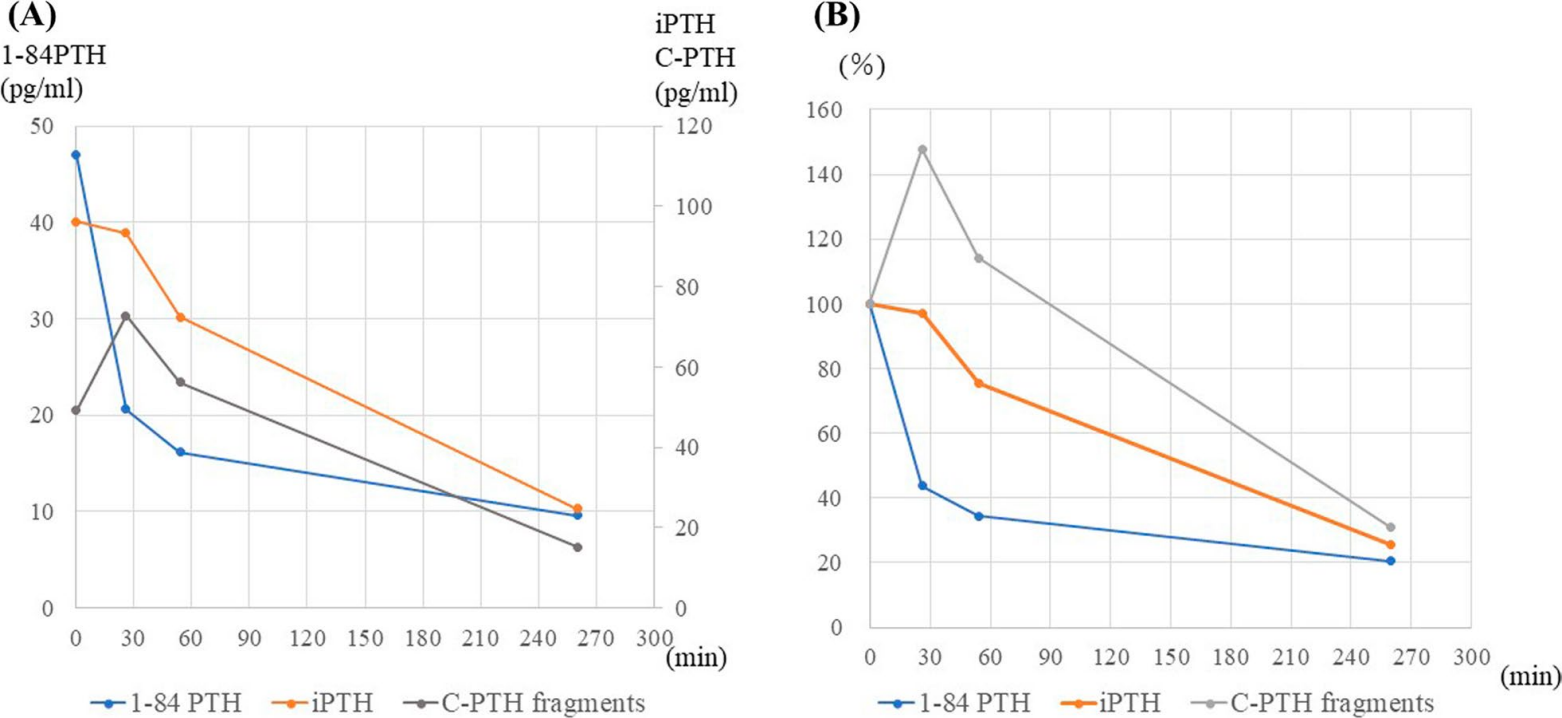
The dynamics of serum concentrations of iPTH, 1–84 PTH, and C-PTH fragments (**A** measured value and **B** % display). IPTH levels were almost unchanged from 96.2 pg/mL preoperatively to 93.3 pg/mL 25 min after its removal. Thereafter, it slowly decreased to a curative level 3 h after removal. The prolonged half-life of C-PTH fragments in this patient likely contributed to the gradual decline in iPTH, as opposed to the more rapid normalization that would have been observed with a 25-min 1–84 PTH measurement. IPTH, intact parathyroid hormone; C-PTH fragments, large carboxyl-terminal parathyroid hormone fragments

The postoperative course was uneventful, and currently, 9 months later, the serum iPTH (44.5 pg/mL) and calcium (2.4 mmol/L) levels were within the normal range. We measured 1–84 PTH using stored serum and concluded that a iPTH spike with a high ratio of C-PTH fragments, which are considered to have a long half-life, caused the slow decline in iPTH. The dynamics of the serum concentrations of iPTH, 1–84 PTH, and C-PTH fragments are shown in Fig. 2. Detailed discussions on the decision to complete surgery despite perceived inadequate iPTH reduction and false-negative results are provided below.

## Discussion

At our institution, iPTH is measured after returning to the recovery room in patients with PHPT whose preoperative diagnosis clearly indicates a single localization of parathyroid adenoma and whose intraoperative findings are consistent with the above findings. Conversely, IOiPTH monitoring is performed in cases where the localization of the pathological glands is uncertain, when multiple gland enlargements are suspected, and in other special cases. In the present case, localization was assessed using ultrasonography and contrast-enhanced CT. Given the negative result of the MIBI test conducted at another hospital and the anticipated challenge in detecting the small lesion within the cyst due to its diminutive parenchyma, a decision was made against repeating the MIBI. Generally, IOiPTH monitoring is not performed if two or more localizations are consistent. Here, although the localization was clear, IOPTH monitoring was performed because cystic parathyroid adenoma is considered very rare [7], and the possibility of FHH could not be completely ruled out. Upon the introduction of IOiPTH assessment at our institution, iPTH measurement was initially conducted 10 min post-removal of a pathologic parathyroid gland [8]. However, due to frequent encounters with false-negative cases, we adjusted the measurement protocol to a minimum of 20 min after removal. The criterion for cure was a PTH level < 40 pg/mL at the last measurement, as suggested by several centers [4, 9, 10]. However, cases of renal impairment were considered individually.

Yang et al. reported that false-negative results with IOPTH may be caused by spikes in PTH levels that occur during adenoma mobilization [3]. Consequently, they modified their protocol to measure IOPTH at the time of cervical incision, when the adenoma was completely removed, and 10 min after removal. In the present case, the IOiPTH levels remained almost unchanged from 96.2 pg/mL preoperatively to 93.3 pg/mL at 25 min after removal of the parathyroid adenoma. This result could indicate either the presence of other pathological glands or the occurrence of a iPTH spike; however, based on the clinical course, we suspected the latter. After an additional 30 min, the IOiPTH levels dropped to 72.3 pg/mL, potentially indicating non-curative surgery [4, 9, 10]. Nonetheless, we completed the surgery due to resolved pressure symptoms, a probable single-gland disease based on preoperative localization, a partial iPTH decrease likely due to special circumstances, and the potential for FHH. With iPTH levels in the ward indicating curability, we measured 1–84 PTH to explore a potential iPTH spike with a high ratio of C-PTH fragments during cystic parathyroid adenoma removal.

Currently, PTH is measured in two main ways: PTH (second-generation), which reacts with 1–84 PTH and C-PTH fragments, and a third-generation assay, which reacts only with 1–84 PTH. We previously reported that C-PTH fragments are directly secreted from parathyroid adenomas and have a longer half-life than 1–84 PTH in patients with PHPT [11]. It has also been reported that C-PTH fragments also originate from the peripheral metabolism of 1–84 PTH [12]. In our previous study, C-PTH fragments constituted < 20% of intact PTH and exhibited approximately twice the half-life in patients with PHPT [11, 13]. The gradual decline in the iPTH levels from 25 to 30 min after removal was attributed to the extremely high ratio of basal C-PTH fragments (78% of PTH fragments in both measurements). The relatively low basal iPTH levels also contributed to the gradual decline in PTH levels, as PTH level decreases more slowly after adenomectomy in patients with PHPT and low basal PTH levels compared to those with high basal PTH levels [14, 15]. This may be due to weak suppression of PTH secretion by other normal parathyroid glands.

The 1–84 PTH assay proved superior to the iPTH assay for IOPTH, displaying a faster decrease in PTH levels and a higher rate of normalization after pathologic parathyroidectomy [11, 13, 16]. Lang et al. reported similar conclusions, emphasizing comparable predictive accuracy between the two methods [17]. In contrast, Gannagé-Yared et al., in a study of 112 patients with PHPT, reported no differences in PTH levels after both assays following parathyroid adenoma removal [17]. They suggested that the discrepancy in results may stem from measurement system variations [18]. In PHPT cases, minimal variation in the ratio of C-PTH fragments to iPTH and blood disappearance rate might limit the advantages of third-generation PTH assays. These ratios are typically elevated with reduced renal function, and in some cases, such as the present case, the C-fragment ratio is quite high, even with normal renal function. In our previous study, the mean ratio of C-PTH to iPTH was 27.5% in patients with PHPT, whereas it was notably higher at 39.6% in individuals with renal failure [13]. Despite the current preference for the 1–84 PTH in IOPTH, it is vital to consider reports stating that the 1–84 PTH assay may not exclusively measure 1–84 PTH under specific circumstances [19]. Considering these factors, a comprehensive approach is necessary, encompassing not only IOPTH criteria but also preoperative localization diagnoses, intraoperative findings, and IOPTH trends.

Our decision not to adopt the 1–84 PTH, despite its acknowledged utility, is rooted in the longer assay time (18 min compared to 10 min for iPTH) and our accumulated data on iPTH in patients with PHPT. Weng reported a slow decline in postoperative PTH levels in two cases of PHPT due to large cystic adenomas, but did not describe detailed PTH dynamics [7]. Therefore, whether the high ratio of C-PTH fragments in our patient with normal renal function was related to the cystic adenoma, and whether there were differences in the ratio of C-PTH fragments and the degree of leakage to blood due to surgical manipulation between the cystic and parenchymal portions is a subject for future studies.

## Conclusions

To our knowledge, this is the first report focusing on the complexities and potential pitfalls of IOPTH monitoring, particularly intraoperative iPTH spikes and disproportionately high concentrations of C-PTH fragments with longer half-lives than those of 1–84 PTH. Factors influencing PTH production and metabolism, as well as assay methods, must be considered, given the absence of criteria predicting IOPTH levels with 100% accuracy.

## Data Availability

Data sharing is not applicable to this article as no datasets were generated or analyzed during the current study.

## Abbreviations

IOPTH: Intraoperative parathyroid hormone
PHPT: Primary hyperparathyroidism
PTH: Parathyroid hormone
IOiPTH: Intraoperative intact parathyroid hormone
C-PTH fragments: Carboxyl-terminal parathyroid hormone fragments
FHH: Familial hypocalciuric hypercalcemia
99mTc-MIBI: 99mTc-methoxy-isobutyl-isonitrile scintigraphy
